# The Light Absorption Enhancement in Graphene Monolayer Resulting from the Diffraction Coupling of Surface Plasmon Polariton Resonance

**DOI:** 10.3390/nano12020216

**Published:** 2022-01-10

**Authors:** Bo Liu, Wenjing Yu, Zhendong Yan, Pinggen Cai, Fan Gao, Chaojun Tang, Ping Gu, Zhengqi Liu, Jing Chen

**Affiliations:** 1 School of Mathematics and Physics, Jiangsu University of Technology, Changzhou 213001, China; cjsliubo@163.com (B.L.); zzhdzjywj@163.com (W.Y.); 2 College of Science, Nanjing Forestry University, Nanjing 210037, China; zdyan@njfu.edu.cn; 3 Center for Optics and Optoelectronics Research, Collaborative Innovation Center for Information Technology in Biological and Medical Physics, College of Science, Zhejiang University of Technology, Hangzhou 310023, China; caippgg@zjut.edu.cn (P.C.); gaofan@zjut.edu.cn (F.G.); 4 College of Electronic and Optical Engineering, Nanjing University of Posts and Telecommunications, Nanjing 210023, China; guping@njupt.edu.cn; 5 College of Physics Communication and Electronics, Jiangxi Normal University, Nanchang 330022, China

**Keywords:** graphene monolayer, absorption efficiency, diffraction coupling, plasmon resonance

## Abstract

In this study, we investigate a physical mechanism to improve the light absorption efficiency of graphene monolayer from the universal value of 2.3% to about 30% in the visible and near-infrared wavelength range. The physical mechanism is based on the diffraction coupling of surface plasmon polariton resonances in the periodic array of metal nanoparticles. Through the physical mechanism, the electric fields on the surface of graphene monolayer are considerably enhanced. Therefore, the light absorption efficiency of graphene monolayer is greatly improved. To further confirm the physical mechanism, we use an interaction model of double oscillators to explain the positions of the absorption peaks for different array periods. Furthermore, we discuss in detail the emerging conditions of the diffraction coupling of surface plasmon polariton resonances. The results will be beneficial for the design of graphene-based photoelectric devices.

## 1. Introduction

When the visible and near-infrared electromagnetic waves are normally incident on the surface of an undoped graphene monolayer suspended in the air, only several percentage points of electromagnetic waves are absorbed by the graphene monolayer. The absorption efficiency (*A*) of graphene monolayer can be estimated by its fine structure constant (α), which is, *A* = πα = π*e*^2^/ℏ*c* ≈ 2.3%. Here, *c* is the light speed, ℏ is the reduced Planck’s constant, *e* is the electron charge, and π is the circumference ratio. The absorption efficiency of 2.3% is a universal value, which does not depend on the wavelength of electromagnetic waves in near-infrared, visible, and even violet regions [1,2]. Due to the considerably good optical and electrical properties, graphene is known to hold a great promising potential in photoelectric devices, such as photodetectors, modulators, perfect absorbers, photovoltaics, photocatalysts, etc. [3,4,5,6,7,8,9,10,11,12,13,14,15,16,17,18]. However, the absorption efficiency of 2.3% is too low for the efficient operation of graphene-based photoelectric devices. Recently, to overcome the difficulty, a number of different solutions have been proposed [19,20], which mainly include surface plasmon polariton resonances [21,22], magnetic resonances [23,24], guided mode resonances [25,26], total internal reflections [27,28], Fabry-Perot resonances [29,30], surface bound states of photonic crystals [31,32], waveguide modes [33], coherent optical beams [34], etc. The fundamental physical principle of these different solutions is to greatly enhance the electric field intensity on the surface of graphene monolayer, and thus improve the absorption efficiency of graphene. In far-infrared and THz regions, the graphene monolayer itself is able to support surface plasmon polariton resonances, which can be utilized to enhance the absorption in graphene [35,36]. However, in the visible and near-infrared regions, the graphene monolayer no longer has this capability, and the other kinds of plasmon resonances in metal nanostructures can be employed to improve the absorption of graphene [37].

The diffraction coupling phenomenon of surface plasmon polariton resonances in the periodic array of metal nanoparticles was theoretically predicted in the early years [38,39], and then it was observed experimentally [40,41]. This diffraction coupling can produce remarkably narrow collective resonances and came to be known as plasmonic surface lattice resonances [42]. In recent years, plasmonic surface lattice resonances have received increasing interest [43], owing to their extremely narrow linewidth and their accompanied great electromagnetic field enhancement. In this experiment, the metal nanoparticle array is commonly prepared on a dielectric substrate. However, it is hard to observe strong surface lattice resonances due to the mismatch in the refractive index of the dielectric substrate and the air [44]. To solve this problem, metal nanoparticles can be lifted by dielectric pillars [45,46] or covered by a dielectric layer with a similar refractive index as the dielectric substrate [47,48]. At present, plasmonic surface lattice resonances are able to provide a large amount of potential applications, such as refractive index sensing [45,46], fluorescent emission [47,48], and tunable lasing [49,50]. To date, there is no report regarding the application of plasmonic surface lattice resonances in improving the light absorption of graphene monolayer.

In this work, we study how to use plasmonic surface lattice resonances to improve the light absorption efficiency of graphene monolayer in the visible and near-infrared wavelength range. The surface lattice resonances result from the collective diffraction coupling effect of localized surface plasmon polariton resonances in gold nanospheres arranged into a periodic array. Through the excitation of surface lattice resonances, the electric fields on the surface of graphene monolayer are considerably enhanced. Therefore, the light absorption in graphene is greatly improved. To well explain the aforementioned physical mechanism, we also employ an interaction model of double oscillators in order to predict the positions of absorption peaks for different array periods. Furthermore, we discuss the emerging conditions of surface lattice resonances. Therefore, our work will be helpful in designing graphene-based photoelectric devices.

## 2. Methods

**Figure 1:**
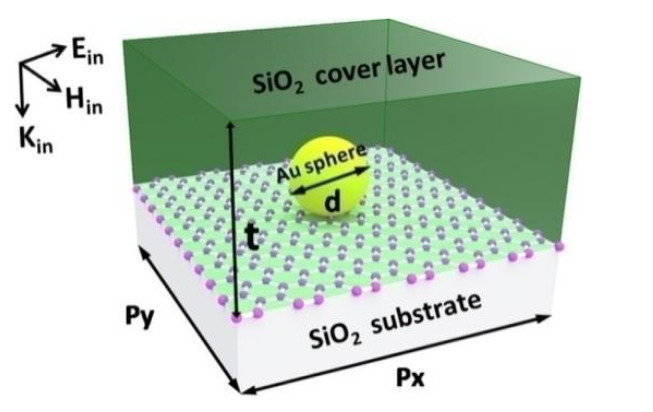
The unit cell of the investigated structure to enhance the light absorption of graphene monolayer.

The investigated nanostructure is schematically shown in Figure 1. The graphene monolayer is placed on the dielectric substrate of SiO_2_. The Au nanospheres on the surface of the graphene monolayer are arranged into periodic arrays, which are covered by the layer of SiO_2_. In this work, we have used a commercial software package “EastFDTD 5.0” (https://www.eastfdtd.com, accessed on 1 September 2013) to calculate the absorption spectra of the graphene monolayer in the visible and near-infrared regions. In addition, some relevant electromagnetic field distributions are calculated by this commercial software package. In our numerical calculations, the Au nanospheres have a complex refractive index, which is dependent on the wavelength of the incident light. The complex refractive index can be obtained from the experimental data [51]. The diameter d of the Au nanospheres is 150 nm. The array periods of the Au nanospheres are p_x_ and p_y_ along the x-axis and the y-axis directions, respectively. Each Au nanosphere is able to support the excitation of the localized surface plasmon polariton resonance [52,53,54,55,56]. The SiO_2_ substrate and the SiO_2_ cover layer have a constant refractive index of 1.45. The thickness *t* of the SiO_2_ cover layer is 500 nm. The most important role played by the SiO_2_ cover layer is to make the surrounding medium of the Au nanospheres homogeneous, and thus realize the diffraction coupling of the localized surface plasmon polariton resonance. The thickness of the graphene monolayer is 0.34 nm. For different wavelengths, the surface conductivity and the anisotropic relative permittivity of the graphene monolayer can be calculated by analytical expressions [57]. In Figure 1, the light is normally incident from top to bottom. The directions of the electric field E_in_, the magnetic field H_in_, and the wave vector K_in_ of the incident light, are indicated by the black arrows in the top left corner. The basic principle of the simulation software is the finite difference time domain method of electromagnetic wave, which is based on the well-known Maxwell equations. In the numerical simulation, we set two perfectly matched layers with a thickness of 500 nm to completely eliminate the reflection of the electromagnetic wave in the positive and negative directions of the *z*-axis. Considering the periodicity of the structure, we also set two periodic boundary conditions in the two directions of the x-axis and the y-axis, respectively. To achieve reliable results with numerical convergence, the mesh size in the regions of graphene is Δs = 0.05 nm, and the mesh size in the other material regions is Δs = 20 nm. Similarly, the time step is set to be Δt = Δs/2c, where c is the speed of light propagating in a vacuum. As the light source, a Gauss pulse with a center wavelength of 900 nm is normally incident on the studied structure. 

## 3. Results and Discussion

**Figure 2:**
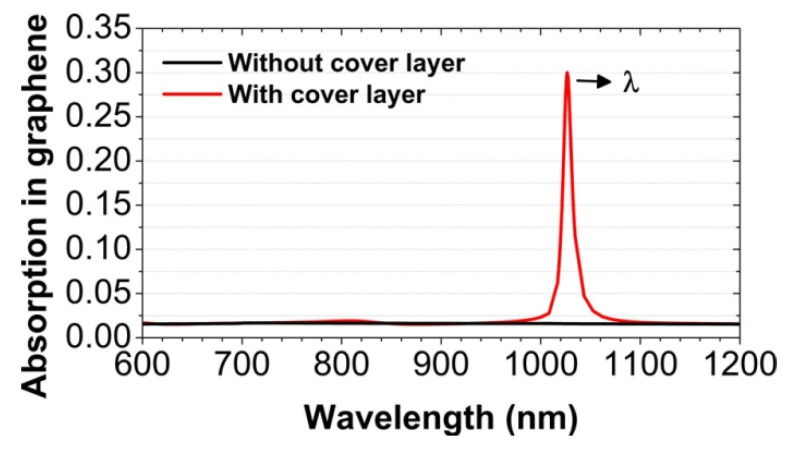
The numerically calculated absorption spectra of the graphene monolayer at normal incidence. Geometrical parameters: The array periods p_x_ = 400 nm and p_y_ = 700 nm; the diameter of the Au nanospheres d = 150 nm; the thickness of the SiO_2_ cover layer t = 500 nm.

In Figure 2, we show the normal-incidence absorption spectra of the graphene monolayer in the visible and near-infrared wavelength range from 600 to 1200 nm, for the two cases: With or without the cover layer of SiO_2_. In the case with the cover layer (see the red line), we observe an obvious absorption peak, which is centered at the wavelength of λ = 1026.5 nm. At the peak, the maximum absorption efficiency of the graphene monolayer can reach up to 30%, which is far larger than the universal value of 2.3%. This light absorption enhancement in the graphene monolayer is beneficial to the graphene-based photoelectric devices. The absorption peak is relatively narrow, which has a full width at half maximum (FWHM) of about 10 nm. The physical mechanism of the absorption peak is the collective diffraction coupling effect of the surface plasmon polariton resonance in the periodic array of Au nanospheres. For comparison, we have also calculated the corresponding absorption spectra in the case without the cover layer. As clearly shown by the black line in Figure 2, the absorption peak will disappear. In the case without the cover layer, the Au nanospheres no longer have a homogeneous surrounding medium. The collective diffraction coupling effect will not happen, due to the mismatch in the refractive index of the dielectric substrate and the air [58]. 

**Figure 3:**
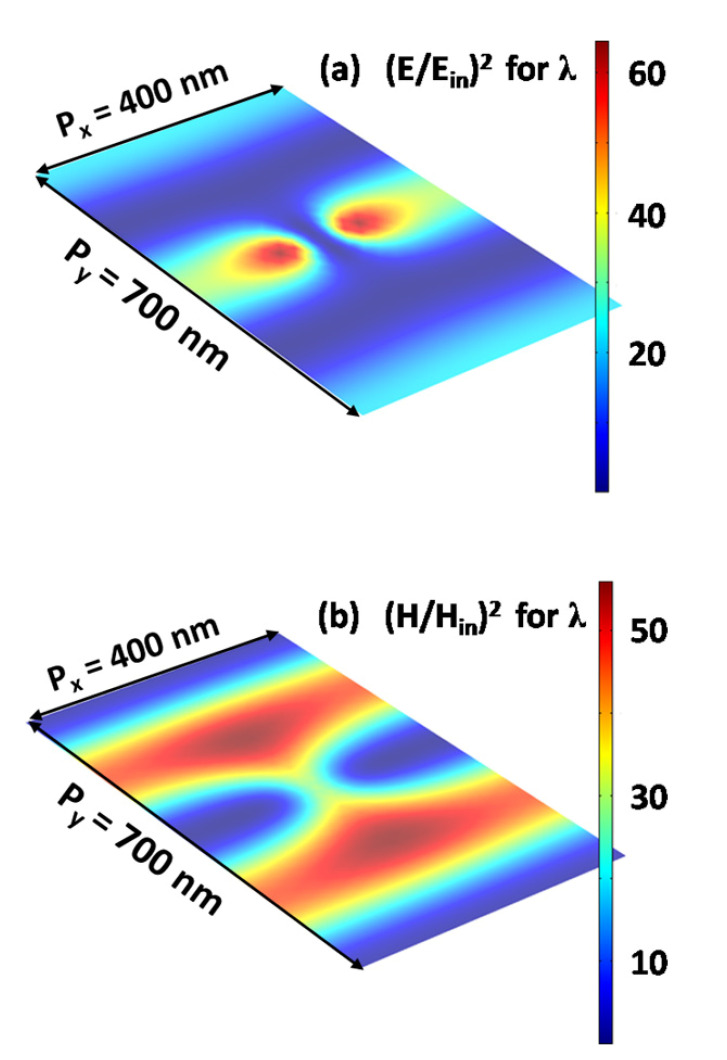
The intensity distributions of the electric fields (**a**) and the magnetic fields (**b**) on the surface of the graphene monolayer at the wavelength of λ = 1026.5 nm.

To effectively understand the physical mechanism of the absorption peak, we calculated the electromagnetic field intensities at the wavelength of λ = 1026.5 nm on the *xy* plane, as shown in Figure 3. Two “hotspots” of electric fields near the Au nanosphere are clearly seen in Figure 3a, which result from the excitation of the localized dipolar plasmon polariton resonance of the Au nanosphere. In addition to the two “hotspots”, there are three parallel belt-shaped regions of the electric field enhancement in Figure 3a, and two parallel belt-shaped regions of the magnetic field enhancement in Figure 3b. These regions of electromagnetic fields are the trails of the diffraction wave propagating along the y-axis direction. In the investigated wavelength range from 600 to 1200 nm, the period p_x_ is only 400 nm. Therefore, all of the diffraction channels along the x-axis direction are closed completely. However, the zero-order diffraction channel along the y-axis direction is open for the period p_y_ = 700 nm. When the zero-order diffraction wave grazes the array of the Au nanospheres, it will strongly interact with the localized dipolar plasmon polariton resonance. This interaction is able to enhance the electric fields on the surface of the graphene monolayer, and thus improve the light absorption efficiency of graphene.

**Figure 4:**
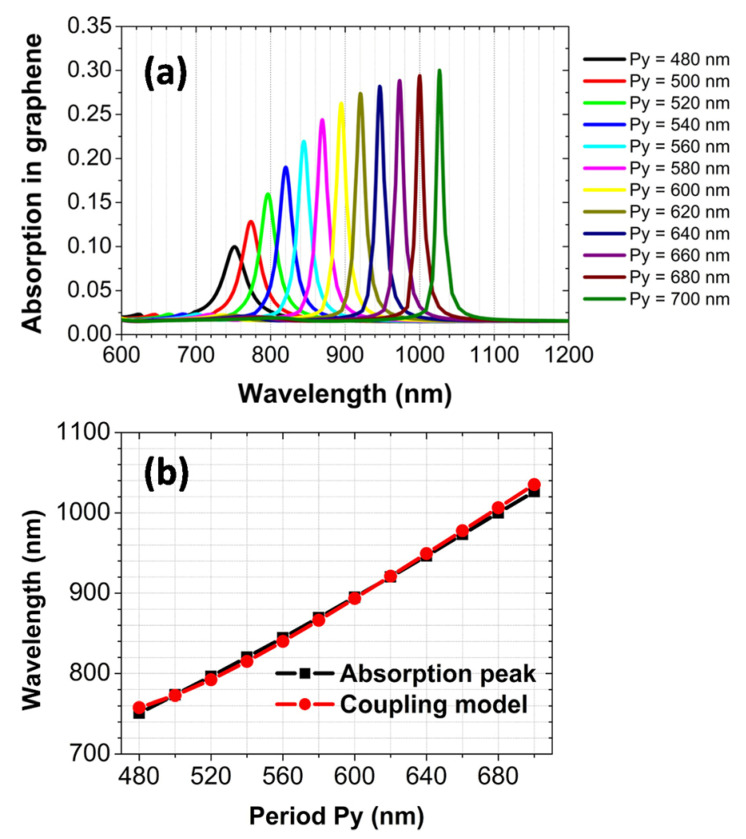
(**a**) The absorption spectra of the graphene monolayer for the different array periods p_y_. (**b**) The dependence of the absorption peak on the array period p_y_.

As discussed above, by properly designing the array periods of the Au nanospheres, the diffraction channel along the x-axis direction can be closed, and only the zero-order diffraction channel along the y-axis direction is open. The zero-order diffraction channel appears near the well-known Wood anomaly, whose wavelength depends on the array period p_y_ (λ_Wood_ = np_y_, *n* = 1.45 is the refractive index of the SiO_2_). Therefore, by changing the array period p_y_, we can tune the position of the absorption peak. In Figure 4a, we have calculated the normal-incidence absorption spectra of the graphene monolayer, when the array period p_y_ is increased from 480 to 700 nm in steps of 20 nm. When the value of p_y_ is increased, it is clearly seen that the absorption peak shifts to the higher wavelength accordingly. To further confirm the physical mechanism of the absorption peak, we have used the interaction model of double oscillators [59] to predict the position of the absorption peak for different array periods p_y_. The black square in Figure 4b gives the practical position of the absorption peak, which is obtained from Figure 4a. The red circle in Figure 4b gives the corresponding position predicted using the interaction model, which is in a good agreement with the practical position. In the interaction model, one oscillator is the localized dipolar plasmon polariton resonance of the Au nanosphere, whose resonance wavelength is 720 nm. The other oscillator is the zero-order diffraction wave along the y-axis direction, whose resonance wavelength is at the Wood anomaly. The interaction strength between these two oscillators is taken as Δ = 25 meV. This value of Δ is chosen to satisfactorily predict the practical position of the absorption peak. The predicted position of the absorption peak is obtained by the following formula:

(1) $$E=(E_{\mathrm{Wood}}+E_{\mathrm{LSP}})/2-\sqrt{\Delta /2+{\left(E_{\mathrm{Wood}}-E_{\mathrm{LSP}}\right)}^{2}/4}$$

where *E*_Wood_ and *E*_LSP_ are the photon energies at the Wood anomaly and the localized dipolar plasmon polariton resonance, respectively. The corresponding wavelength of the photon energy *E* is the predicted position of the absorption peak.

**Figure 5:**
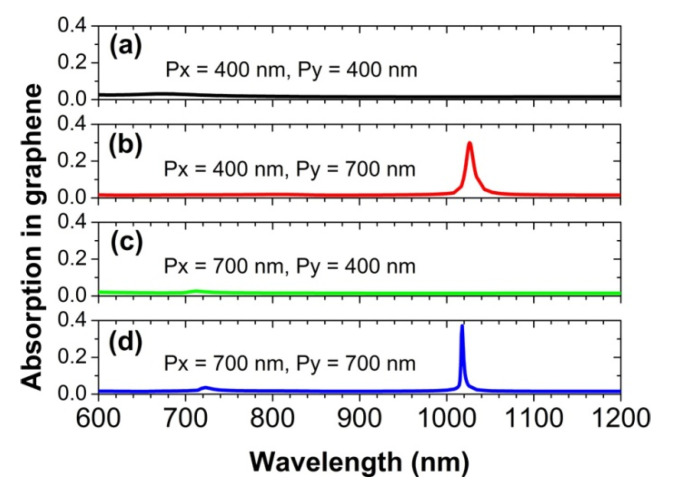
The calculated absorption spectra of the graphene monolayer for different array periods at normal incidence.

Here, we will discuss the emerging conditions of the diffraction coupling of localized dipolar plasmon polariton resonance in the periodic nanosphere array. To achieve a strong collective effect of diffraction coupling, the surrounding medium of the Au nanospheres should be homogeneous, and the cover layer of SiO_2_ plays this role, as shown in Figure 1. In addition to this necessary condition, some other conditions should be satisfied, as well. First, the period of the nanosphere array should be large enough for the zero-order diffraction channels to be open in the investigated wavelength range from 600 to 1200 nm. If the period is too small, the collective effect of diffraction coupling will not happen. As clearly seen in Figure 5a, the absorption peak of the graphene monolayer disappears, when the array period p_y_ is also shortened to be 400 nm. However, when the array period p_y_ is set to be 700 nm, the absorption peak will appear, as shown in Figure 5b. Second, the propagation direction of the diffraction wave must be perpendicular to the polarization direction of the localized dipolar plasmon polariton resonance. In all of the cases studies in this work, the electric field of the incident light is always along the x-axis direction. Therefore, the polarization direction of the localized dipolar plasmon polariton resonance also remains along the x-axis direction. For the array periods of p_x_ = 400 nm and p_y_ = 700 nm, the diffraction wave propagates along the y-axis direction. In this case, the propagation direction of the diffraction wave and the polarization direction of the localized dipolar plasmon polariton resonance are mutually perpendicular. The above two conditions are satisfied simultaneously. Therefore, we can observe an absorption peak in Figure 5b. In contrast, for the array periods of p_x_ = 700 nm and p_y_ = 400 nm, the diffraction wave propagates along the x-axis direction, whose propagation direction is parallel to the polarization direction of the localized dipolar plasmon polariton resonance. For this situation, the second condition is not satisfied. Therefore, in Figure 5c, we could not observe an absorption peak. In Figure 5d, with the array periods of p_x_ = p_y_ = 700 nm, the zero-order diffraction channels along the x-axis direction and the y-axis direction are both open, and the second condition is also satisfied for the diffraction wave propagating along the y-axis direction. As a result, the absorption peak can exist, as well.

**Figure 6:**
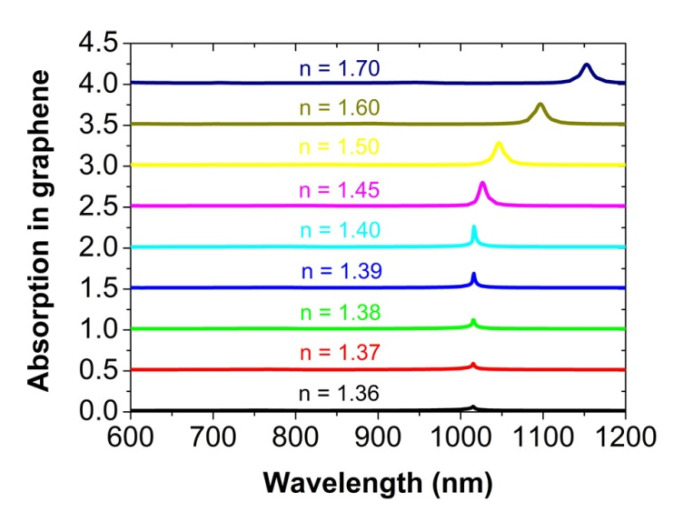
The calculated absorption spectra of the graphene monolayer for the different refractive index n of the cover layer at normal incidence. For clarity, the above absorption spectrum is vertically offset by 0.5 from the one below.

Next, we will consider the influences of some factors on the absorption peak of the graphene monolayer. Figure 6 shows the effect of a refractive index mismatch between the dielectric substrate and the covering medium. When the refractive index n of the covering medium decreases from 1.45 to 1.36, the light absorption efficiency of the graphene monolayer will be reduced quickly from 30% to about 6.5%. For the refractive index n of the covering medium to be increased from 1.45 to 1.70, the absorption of the graphene monolayer will also be reduced slowly to about 24%. The refraction index mismatch results in different phase velocities for the diffractive wave to propagate above and below the substrate [41,42]. If a very large difference in the refractive index is found between the cover layer and the dielectric substrate, it is hard to realize a strong interaction between the diffractive wave and the localized dipolar plasmon polariton resonances of Au nanospheres [44]. As a result, the plasmonic surface lattice resonance mode in the metal nanoparticle arrays could not be formed, and the absorption peak of the graphene monolayer will also disappear. In addition, with the increasing value of the refractive index n, the absorption peak of the graphene monolayer will red-shift, since the wavelength of the Wood anomaly becomes longer. At the same time, the bandwidth of the absorption peak will be broader, due to the larger radiation decay of the plasmonic surface lattice resonance mode.

**Figure 7:**
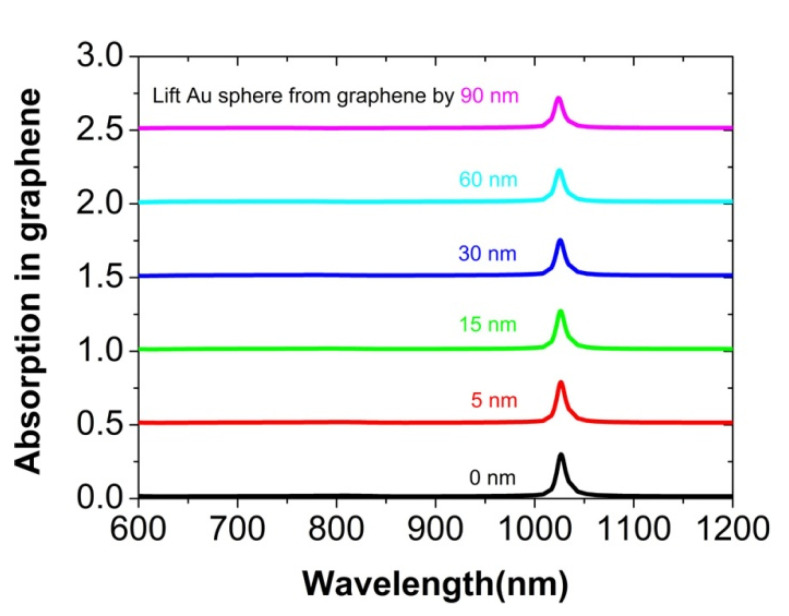
The calculated absorption spectra of the graphene monolayer at normal incidence, when the Au nanosphere is lifted from the graphene by different distances. For clarity, the above absorption spectrum is vertically offset by 0.5 from the one below.

**Figure 8:**
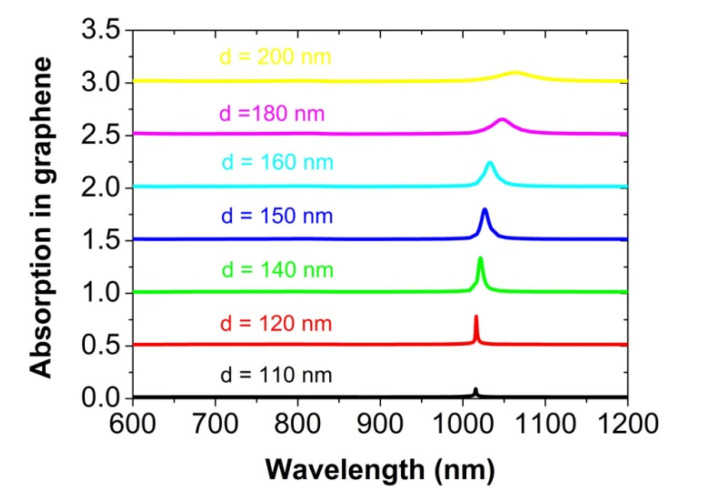
The normal-incidence absorption spectra of the graphene monolayer for the different diameters d of the Au nanosphere. For clarity, the above absorption spectrum is vertically offset by 0.5 from the one below.

The distance between the graphene and the nanosphere array is another factor to affect the light absorption of the graphene, as shown in Figure 7. When the Au nanosphere is lifted from the graphene monolayer by 5 to 90 nm, the absorption peak will decrease gradually to 22%, since the plasmonic near-field near the graphene becomes weak. Moreover, we have studied the influence of the size of the Au nanosphere on the absorption peak, which is shown in Figure 8. For the diameter d of the Au nanosphere to be about 160 nm, the graphene has a maximum absorption of about 33% at the plasmonic surface lattice resonance. When the diameter d increases to 200 nm, the peak value will be reduced to about 10%. In this case of larger diameter, the Au sphere array reflects more incident light, and thus reduces the absorption in graphene. If the diameter *d* is only 110 nm, the absorption peak will almost not exist, since the diffraction coupling of surface plasmon polariton resonance among the Au nanospheres is very weak [44]. It is well known that the absorption and the scattering of an individual nanosphere are radius-dependent, which are directly proportional to r^3^ and r^6^, respectively, in the long-wave limit, in which the wavelength of the incident light is far larger than the size of a nanosphere [60]. In our work, this radius-dependent relation is not found, since the long-wave limit condition is not well satisfied and simultaneously there is a strong interaction among the Au nanospheres. At the plasmonic surface lattice resonance, the absorption of the Au nanospheres is about 12%, but it is very low for other wavelengths from 600 to 1200 nm.

**Figure 9:**
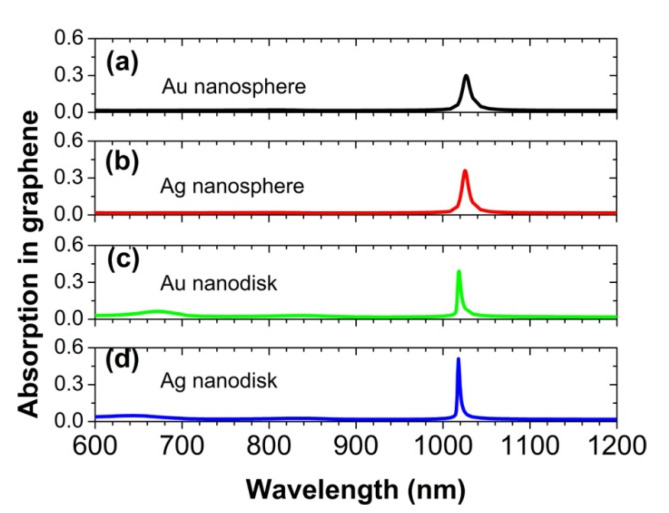
The normal-incidence absorption spectra of the graphene monolayer, for the Au nanosphere to be replaced by the Ag nanosphere, the Au nanodisk, and the Ag nanodisk, respectively. The array periods are px = 400 nm and py = 700 nm. The nanospheres have a diameter of 150 nm. The nanodisks have a diameter of 120 nm and a thickness of 60 nm.

In Figure 9, we have also studied the effects of different metal materials and different nanoparticle shapes on the absorption peak of graphene. When the Au nanosphere is replaced by an Ag nanosphere, the absorption peak becomes a little sharper and increases slightly to 36%, owing to the relatively smaller Joule loss in the Ag material. For the Au nanosphere to be replaced by Au nanodisk or Ag nanodisk, the absorption peak of graphene becomes much narrower, which is able to reach 39% and 51%, respectively. This proves that the proposed physical mechanism is universal. The key role played by the periodical array of metal nanoparticles is to excite the plasmonic surface lattice resonance, which can greatly enhance the electric fields on the surface of graphene, and thus improve the light absorption efficiency of the graphene monolayer. Finally, we should mention that the two oscillator models are still applicable to the above modified parameters. However, the excitation wavelength of the surface plasmon polariton resonance of metal nanoparticles will change for different parameters. Similarly, the interaction strength Δ will also change, which must be reset to accurately predict the position of the absorption peak.

## 4. Conclusions

In summary, we have investigated a physical mechanism for enhancing the light absorption efficiency of graphene monolayer from the universal value of 2.3% to about 30% in the visible and near-infrared wavelength range. The physical mechanism is based on the collective diffraction coupling effect of localized surface plasmon polariton resonance in the periodic array of metal nanoparticles. Through this collective diffraction coupling effect, the electric fields on the surface of graphene monolayer are considerably enhanced, and thus the light absorption efficiency of graphene monolayer is greatly improved. To further confirm the physical mechanism, we have used the interaction model of double oscillators to predict the positions of the absorption peaks for different array periods. Moreover, we have discussed in detail the emerging conditions of the collective diffraction coupling effect. First, the array period should be large enough for the zero-order diffraction channels to be open in the investigated wavelength range. Second, the propagation direction of the diffraction wave must be perpendicular to the polarization direction of the localized dipolar plasmon polariton resonance. In addition, for the collective diffraction coupling effect to take place, the surrounding medium of the metal nanoparticle array should be homogeneous. The above results are beneficial for the design of the graphene-based photoelectric devices.

## Data Availability

The data presented in this study are available on request from the first or corresponding author.
